# Effects of Exhaustive Exercise on Adiponectin and High-Molecular-Weight Oligomer Levels in Male Amateur Athletes

**DOI:** 10.3390/biomedicines12081743

**Published:** 2024-08-02

**Authors:** Marta Mallardo, Ester Tommasini, Sara Missaglia, Claudio Pecci, Ermanno Rampinini, Andrea Bosio, Andrea Morelli, Aurora Daniele, Ersilia Nigro, Daniela Tavian

**Affiliations:** 1 Dipartimento di Medicina Molecolare e Biotecnologie Mediche, Università degli Studi di Napoli “Federico II”, Via Pansini, 80131 Napoli, Italy; marta.mallardo@unicampania.it; 2 CEINGE Biotecnologie Avanzate “Franco Salvatore” Scarl, Via G. Salvatore 486, 80145 Napoli, Italy; ersilia.nigro@unicampania.it; 3 Laboratory of Cellular Biochemistry and Molecular Biology, CRIBENS, Università Cattolica del Sacro Cuore, 20145 Milan, Italy; ester.tommasini@unicatt.it (E.T.); sara.missaglia@unicatt.it (S.M.); daniela.tavian@unicatt.it (D.T.); 4 Department of Psychology, Università Cattolica del Sacro Cuore, 20123 Milan, Italy; 5 Human Performace Laboratory, MAPEI Sport Research Centre, 21057 Olgiate Olona, Italy; claudio.pecci@mapeisport.it (C.P.); ermanno.rampinini@mapeisport.it (E.R.); andrea.bosio@mapeisport.it (A.B.);; 6 Sport and Exercise Discipline Group, Human Performance Research Centre, Faculty of Health, University of Technology Sydney, Moore Park, Sydney, NSW 2021, Australia; 7 Dipartimento di Scienze e Tecnologie Ambientali, Biologiche, Farmaceutiche, Università della Campania “Luigi Vanvitelli”, Via A. Vivaldi, 81100 Caserta, Italy

**Keywords:** physical exercise, adiponectin, HMW oligomers, VO_2peak_, Power_peak_

## Abstract

Physical activity promotes metabolic health and prevents lifestyle-related diseases. Adiponectin is specifically produced by adipose tissue and comes in three forms, differing in terms of weight: trimers (LMW), hexamers (MMW), and high-molecular-weight (HMW) oligomers. The oligomers are associated with the beneficial effects of adiponectin. In this study, we aimed to investigate the impact of a single bout of exhaustive exercise on adiponectin expression in 25 male amateur athletes, divided into two groups, one comprising young adults (YAs) (*n* = 15), and the other comprising middle-aged adults (MAs) (*n* = 10). Body fat was estimated through skinfold thickness. Adiponectin levels were assessed at baseline and at 15 min and 24 h post-exercise, while HMW oligomer levels were evaluated at baseline and at 24 h post-exercise. We observed a significant increase in total adiponectin at both 15 min and 24 h after exercise, with there being a more evident effect among the YA subjects. HMW oligomers also increased significantly after exercise both in the total sample and among the YA subjects, but this was not the case in the MA subjects. The increase in adiponectin levels was significantly associated with Power_peak_. Furthermore, a significant inverse correlation was found between basal adiponectin and VO_2peak_ and Power_peak_. In conclusion, a single bout of exhaustive exercise can rapidly and significantly enhance the basal circulating adiponectin concentration, which seems to be negatively associated with maximal aerobic capacity.

## 1. Introduction

Physical activity and exercise are known to play an essential role in improving physical fitness and overall health [1,2]. They are widely recognized as effective preventive and therapeutic measures for a range of diseases. In particular, moderate and regular physical exercise appear to be a very effective tool in combating metabolic diseases related to obesity. Indeed, several recent studies have shown that high-intensity exercise, alone or combined with strength exercises, improves metabolic, immune, and inflammatory functions [3,4]. In particular, it has been observed that, following acute physical exercise, the levels of Interleukin 6 and Interleukin 10 increase, while the expression of tumor necrosis factor alpha is reduced [5,6].

There are several scientific studies available in the literature on the variations in the expression of numerous cytokines following physical protocols. These data demonstrate that many organs and tissues, through the secretion of cytokines, participate in the physiological adaptations that occur during physical exercise [5]. Adipose tissue (AT) also secretes various adipocytokines, which participate in the regulation of energy metabolism and inflammation [7,8,9,10]. In fact, the literature data suggest the involvement of AT endocrine function, as evidenced by the change in the expression of adipokines, including adiponectin [11]. Adiponectin, a polypeptide of 244 amino acids, is synthesized and released specifically by adipose tissue such as oligomers of different molecular weights, namely trimers (low molecular weight, LMW), hexamers (medium molecular weight, MMW), and high-molecular-weight (HMW) oligomers [12,13]. Adiponectin is an endocrine mediator involved in improving insulin sensitivity and energy metabolism [12]. The literature data have shown that HMW oligomers are the most active form of this adipocytokine, being involved in the regulation of body weight and energy balance [14]. Often measured in studies involving obese subjects, adiponectin has been inversely linked to BMI (body mass index), body fat content, and inflammation [12]. Most studies have reported that serum adiponectin concentrations tend to rise with age in healthy males and females [15,16], but a few studies have found a decrease [16] or no change [17]. In an extremely large population, Obata et al. also found a significant positive association between serum adiponectin levels and age in healthy subjects [14].

Some research suggests that acute exercise, as well as regular exercise, can lead to higher levels of adiponectin in the blood [18,19,20,21,22,23,24]. Previously, we investigated basal total serum adiponectin expression and its oligomeric status in professional water polo players and in healthy sedentary controls, finding higher levels of serum adiponectin in the control group and no difference between the two groups in terms of HMW oligomer status [25]. The implications of the relationship between exercise and adiponectin levels remain a subject of debate. Moreover, the exact biological mechanism through which regular exercise affects adiponectin levels remains to be fully understood.

Therefore, since data on the effects of acute exercise on total and HMW adiponectin oligomers in healthy and active subjects are limited, further studies are needed.

Against this backdrop, the hypothesis underlying our study is that adiponectin levels, both total and HMW oligomers, might be influenced by a single bout of exhaustive exercise and that such regulation might be related to exercise capacity.

To investigate this hypothesis, we recruited 25 male amateur athletes, divided into two groups based on age—a young adult group (YA) (*n* = 15) and a middle-aged adult group (MA) (*n* = 10)—who were tasked with performing incremental exhaustive exercise on a cycle ergometer. Serum concentrations of adiponectin were assessed at baseline, 15 min post-exercise, and 24 h post-exercise, whereas HMW oligomers were evaluated at baseline and 1 day after exercise. We compared the effects of this exercise by analyzing the responses of the young and middle-aged amateur athletes. Adiponectin was correlated with anthropometric, body composition, and performance parameters.

## 2. Materials and Methods

### 2.1. Participants

Twenty-five healthy male individuals (aged 20–65 years, actively participating in amateur sports and training regularly 2–3 times per week) were voluntarily enrolled in the study. All participants were amateur athletes, of which 17 partake in individual sports (i.e., cycling and boxing), and 8 partake in team sports (i.e., soccer, rugby, and martial arts).

The Italian version of the International Physical Activity Questionnaire was used to evaluate the physical activity levels of the participants [26,27]. Moderately active and highly active individuals with a body mass index (BMI) ≤ 30 kg/m^2^ were involved in the study. The exclusion criteria were self-reported history of cardiovascular diseases, neurological disorders, musculoskeletal impairment, metabolic syndrome, obesity, renal disease, respiratory diseases, and inflammatory/autoimmune diseases.

The amateur athletes were divided into two age groups: a young adult group (YA) (*n* = 15, 25.3 ± 4.1 years) and a middle-aged adult group (MA) (*n* = 10, 54.0 ± 5.9 years).

They were informed of the aim and nature of the study, as well as the practical details and possible risks associated with the study. This study conforms to the ethical principles outlined in the Declaration of Helsinki. The Ethics Commission of the Università Cattolica del Sacro Cuore of Milan approved all the study procedures (protocol number: 28-22; approval date: 4 May 2022). Written informed permission for personal data treatment and biochemical analysis was obtained from all participants. Participants completed all the assessments over two consecutive days.

### 2.2. Sample Collection, Anthropometric, and Body Composition Parameters

At the same time of the day, from 10:00 a.m. to 1:00 p.m., participants were asked to arrive at the laboratory after 2 h of fasting and 48 h of abstention from moderate and/or vigorous physical activity28. Serum samples were collected before exercise, 15 min after reaching exhaustion, and 24 h after reaching exhaustion.

The analysis of anthropometric and body composition parameters included measurements of weight and height, BMI calculation, and the determination of each participant’s fat mass (FM) percentage through the skinfold thickness, as previously reported [26,27].

### 2.3. Exercise Protocol

Maximal aerobic capacity was determined using a cardiopulmonary exercise test. An incremental exercise test designed to induce volitional exhaustion on a cycle ergometer (LC6 Monark; Vansbro, Sweden and Excalibur Sport, Lode BV, Groningen, The Netherlands) was performed to measure peak oxygen uptake (VO_2peak_), peak power (Power_peak_), and peak heart rate (HR_peak_). After 2 min of resting oxygen uptake assessment, the test started at a power output of 80 W, with this being increased by 20 W every minute. Respiratory gasses (Quark CPET, COSMED, Rome, Italy, and Vyntus CPX, Vyaire GmbH, Hochberg, Germany) and heart rate (Garmin, Olathe, KS, USA) were measured continuously during cycling.

### 2.4. ELISA

The concentration of total serum adiponectin was measured with an enzyme-linked immunosorbent assay (ELISA) using a polyclonal antibody produced in-house versus a human adiponectin amino acid fragment (H2N-ETTTQGPGVLLPLPKG-COOH) as previously described [25]. Each sample was tested three times in duplicate. Additionally, the circulating HMW adiponectin was measured with a specific human ELISA kit according to the manufacturer’s instructions (R&D Systems, Minneapolis, MN, USA).

### 2.5. Western Blotting

Five microliters of serum were treated with 1 × Laemmli buffer, heated at 95 °C for 2 min, and loaded under non-reducing conditions on 10% SDS-PAGE gel and transferred as previously described [15]. The blots were scanned using the ChemiDoc MP imaging system (Bio-Rad, Hercules, CA, USA) and analyzed by carrying out densitometry with ImageJ software V 1.53.

### 2.6. Statistical Analysis

Statistical analysis was conducted using SPSS software (Version 29). The Shapiro–Wilk test was employed to assess the normality of the data distribution. Data from the total sample met the normality assumptions, and power analyses ensured sufficient statistical power for carrying out parametric tests. Therefore, the effect of time was analyzed by using a repeated-measures ANOVA, and post hoc contrasts were conducted to identify significant differences between means of time points in the total sample. Regarding data from the age groups, given the small sample size and associated lack of statistical power within the age groups, non-parametric tests were utilized. Specifically, the Friedman test for repeated measures was employed to assess differences within groups, with Bonferroni correction applied. Additionally, the Mann–Whitney U test was used to test differences between groups (YA and MA). To determine the association between variables, Pearson’s correlation analysis was performed. Partial correlation adjusted for age was performed to eliminate the strongest confounder. The level of significance was set at 0.05. The variation in adiponectin (delta) between baseline and post-exercise was computed (delta = post-exercise adiponectin level − baseline adiponectin level).

## 3. Results

### 3.1. Anthropometric Characteristics and Physical Exercise Parameters of Study Participants

Twenty-five male amateur athletes completed the study. The characteristics of the participants are shown in Table 1.

No significant differences in terms of BMI between YAs and MAs were found. The FM percentage results were significantly lower for YAs compared to MAs. However, according to the normative fitness categories for body fat outlined by the ACSM [28], the YA subjects have a good body composition, while the MA subjects have an excellent body composition. Regarding physical exercise parameters, YAs reached significantly higher HR_peak_ values but lower Power_peak_ values during the maximal incremental exercise protocol. No significant differences in relative VO_2peak_ were found. Nevertheless, when compared against the ACSM normative fitness categories for VO_2max_ [28], the cardiorespiratory fitness of YAs was deemed satisfactory, while that of MAs was classified as excellent.

### 3.2. Total Serum Adiponectin Levels Increase after Acute Exercise

**Figure 1:**
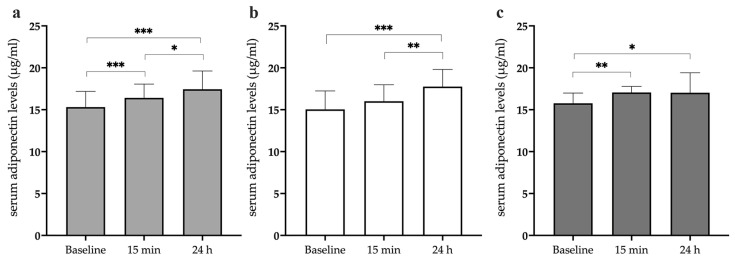
Comparison of serum adiponectin levels at baseline, at 15 min following a single bout of exhaustive exercise, and at 24 h following a single bout of exhaustive exercise among the 25 participants. *p*-values were obtained using an ANOVA for repeated measures to compare baseline and post-single-bout-of-exhaustive-exercise adiponectin levels (**a**). Serum adiponectin levels at baseline, at 15 min post-exercise, and at 24 h post-exercise in young adults (YAs) (**b**) and middle-aged adults (MAs) (**c**). The Friedman test was used to verify differences within groups. Data are expressed as means ± SD. * *p* < 0.05; ** *p* < 0.01; *** *p* < 0.001.

To investigate the change in circulating adiponectin levels in response to exhaustive exercise, we first analyzed total serum adiponectin at baseline, at 15 min, and at 24 h post-exercise. Interestingly, we observed a significant increase in adiponectin concentration at both 15 min (*p* < 0.001) and 24 h (*p* < 0.001) after a single bout of exhaustive exercise compared to at baseline. A significant elevation was also noted from 15 min to 24 h post-exercise (*p* = 0.016) (Figure 1a). Indeed, adiponectin levels increased from the basal level of 15.3 ± 1.9 μg/mL to 16.4 ± 1.6 μg/mL at 15 min post-exercise and to 17.5 ± 2.2 μg/mL at 24 h post-exercise.

Subsequently, when the study population was divided into two subgroups according to age, YAs (below 35 yrs) and MAs (above 46 yrs), the increase in adiponectin levels was similar between the two groups. In the YA group, the increase in adiponectin levels from baseline was noticeable only one day after exercise (*p* < 0.001) and from 15 min to 24 h post-exercise (*p* = 0.002). No significant increase was observed immediately after exercise (baseline: 15.03 ± 2.2 μg/mL; 15 min post-exercise: 16 ± 2 μg/mL; 24 h post-exercise: 17.8 ± 2.1 μg/mL) (Figure 1b). Within the MA group, basal adiponectin levels of 15.8 ± 1.2 µg/mL increased significantly at both time points following exercise (15 min post-exercise: 17.05 ± 0.7 µg/mL (*p* = 0.007); 24 h post-exercise: 17.02 ± 2.4 µg/mL (*p* = 0.044) (Figure 1c). As for the comparison between age groups, no differences were found between MAs and YAs at each evaluated time point (baseline, *p* = 0.346; 15 min, *p* = 0.108; 24 h, *p* = 0.405). Supplementary Figure S1 shows the adiponectin levels of each enrolled subject for each time point.

### 3.3. Adiponectin Levels Correlate with VO_2peak_ and Power_peak_ Following Acute Exercise

Subsequently, we investigated the potential relationships between baseline adiponectin and BMI, FM, VO_2peak_, and Power_peak_ in the entire study population. As shown in Table 2, there were significant negative correlations between baseline adiponectin and both VO_2peak_ and Power_peak_. Moreover, these parameters withstood adjustment for age in partial correlation analysis. Within the participants, dividing them into YAs and MAs, we found a significant negative correlation between baseline adiponectin and Power_peak_ in the YA group (Table 3). In the MA group, we found no significant correlation with any of the analyzed parameters, probably due to the lower number of analyzed subjects. After adjusting for age, significant correlations persisted between baseline adiponectin and both Power_peak_ and VO_2peak_ in YAs. Interestingly, following partial correlation adjustment, a significant correlation between baseline adiponectin and Power_peak_ was also observed in MAs (Table 4). A significant and positive correlation was found between the increase in adiponectin levels from baseline to 15 min post-exercise and Power_peak_ in the total sample (r = 0.428; *p* = 0.033). No other correlations were found between the increase in adiponectin levels and participant characteristics in both the total sample and the two individual age groups.

### 3.4. HMW Adiponectin Oligomers Increase after a Single Bout of Exhaustive Exercise

**Figure 2:**
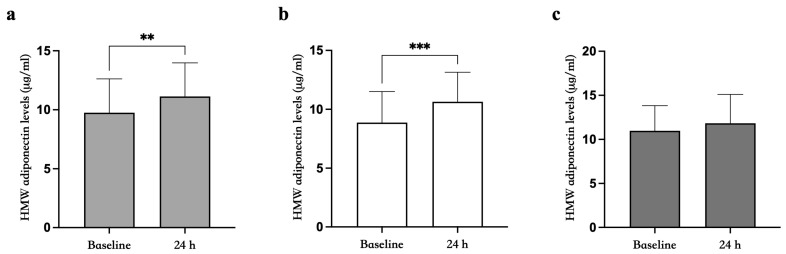
Comparison of HMW adiponectin levels at baseline and 24 h following a single bout of exhaustive exercise in the 25 participants. (**a**) HMW adiponectin levels at baseline and 24 h post-exercise in young adults (YAs) (**b**) and middle-aged adults (MAs) (**c**). The Friedman test was used to verify differences within groups. Data are expressed as means ± SD. ** *p* < 0.01; *** *p* < 0.001.

Since data from the literature demonstrate that HMW oligomers are the most active form of adiponectin, we analyzed them to investigate whether the increase in total serum adiponectin we observed was primarily related to the increase in HMW oligomers. Therefore, we analyzed HMW adiponectin at baseline and 24 h post-exercise using both ELISA and Western blotting (WB). Interestingly, considering the entire population, HMW adiponectin levels significantly increased from the basal level of 9.7 ± 2.9 μg/mL to 11.1 ± 2.8 μg/mL 24 h post-exercise (*p* < 0.01) (Figure 2a). Subsequently, when the study population was divided into two age groups, the increase in HMW adiponectin levels was significant in the YA group (baseline: 8.9 ± 2.6 μg/mL; 24 h post-exercise: 10.6 ± 2.5 μg/mL (*p* < 0.001)) (Figure 2b) but not in the MA group (baseline: 10.9 ± 2.8 μg/mL; 24 h post-exercise: 11.8 ± 3.3 μg/mL) (Figure 2c). Analogously to the findings for the total serum adiponectin levels, no significant difference was found between the two groups for HMW oligomers at baseline (*p* = 0.128) and at 24 h (*p* = 0.380) post-exercise.

**Figure 3:**
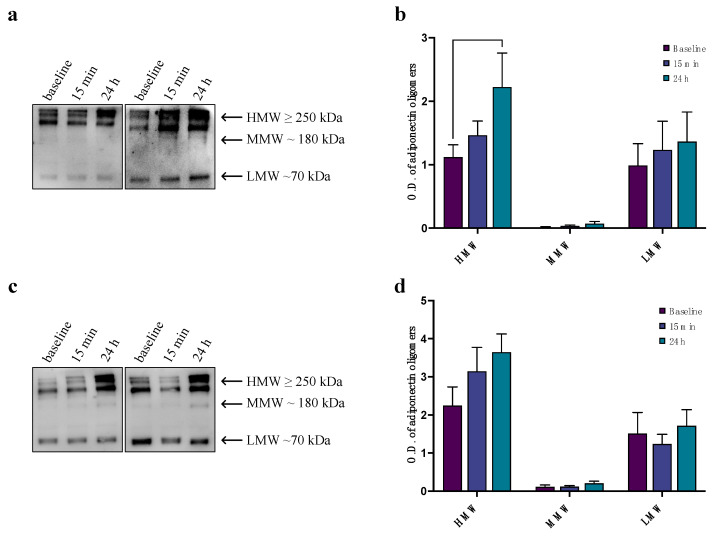
Western blotting analysis of adiponectin oligomers in sera from amateur athletes at baseline, at 15 min following a single bout of exhaustive exercise, and at 24 h following a single bout of exhaustive exercise. (**a**) A representative image of the Western blot oligomeric distributions [HMW (≥250 kDa), MMW (180 kDa), and LMW (70 kDa)] of two YA subjects at baseline, at 15 min post-CPET, and at 24 h post-CPET. (**b**) Graphical representation of pixel quantization of all analyzed YA subjects included in the study was performed via densitometric analysis, carried out with ImageLab software V 1.53; *p* < 0.01. (**c**) A representative image of the Western blot oligomeric distributions [HMW (≥250 kDa), MMW (180 kDa), and LMW (70 kDa)] of two MA subjects at baseline, at 15 min post-exercise, and at 24 h post-exercise. (**d**) Graphical representation of pixel quantization of all analyzed MA subjects included in the study was performed via densitometric analysis, carried out with ImageLab software V 1.53; *p* < 0.01.

The results of our WB analysis confirmed that adiponectin levels increased 24 h after exercise in the entire study population. The results of the densitometric analysis (Figure 3b,d) highlight a specific increase in HMW oligomers (Figure 3a,c). When considering participants divided on the basis of age, the increase in HMW oligomers was significant only in the YA group (Figure 3b).

## 4. Discussions

The main findings of the current study were a significant increase in adiponectin concentrations, predominantly due to an increase in HMW oligomers. Furthermore, the analysis of the correlation among the adiponectin values and the main physical exercise parameter indicates that the baseline adiponectin levels correlate with VO_2peak_ and Power_peak_.

It is widely recognized that physical exercise is essential for maintaining and enhancing health through the modulation of specific cytokine concentrations [5]. Adipose tissue also secretes various adipocytokines, which play a role in regulating energy metabolism and inflammation [7,8,9,10]. In this study, we observed that adiponectin is highly responsive to acute exhaustive exercise. In particular, a bout of acute exercise results in a significant increase in serum adiponectin levels at both 15 min and 24 h post-exercise, with the highest levels observed 1 day after the end of exercise. Our results align with prior research indicating that high-intensity exercise leads to a rapid rise in adiponectin levels among trained athletes [29,30]. Kraemer et al. demonstrated that well-trained runners performing strenuous running at 60% to 100% of their VO_2max_ exhibited a small (10%) but significant increase in post-exercise adiponectin [31]. Similarly, Jamurtas et al. demonstrated that a mean increase in adiponectin concentration of approximately 15% occurred after the first 30 min of recovery from water sculling at 75% VO_2max_ for approximately 30 min [32]. Similarly, another study also demonstrated a delayed increase in adiponectin after the first 30 min recovery period following maximal 6000 m ergometer rowing (∼20 min) [30]. How different types of protocols might impact adiponectin regulation is still a matter of debate. Our findings suggest a novel insight, implying that this increase is not only immediate but also long-lasting. Future research is needed to understand the molecular mechanisms behind this result.

It should also be underlined that adiponectin is a complex protein made by oligomers of different molecular weights and that HMW oligomers represent its activated form, essential for the metabolism of glucose and lipids [17]. The precise mechanisms responsible for the secretion of HMW oligomers remain unclear; however, they are known to play a critical role in mediating the beneficial effects of adiponectin [13]. Our findings demonstrated an increase in high-molecular-weight (HMW) oligomer concentrations 24 h after a bout of acute exercise. These results were further confirmed by Western blotting analysis, which indicated elevated levels of HMW oligomers one day after exercise. On the contrary, Numao et al. reported no changes in HMW levels during and after acute moderate-intensity aerobic exercise in healthy young men [33]. Notably, there are no studies reporting data on total/HMW adiponectin concentrations after a single bout of exhaustive exercise. Most of the studies in the literature on HMW oligomer levels have assessed the chronic effect of training programs lasting more than 7 days on HMW oligomers, with conflicting results being reported [34,35,36].

In agreement with most previous studies, we detected lower levels of total serum adiponectin in young males compared to middle-aged healthy males under basal conditions [13,14], but we failed to find a significant difference between the two groups, probably due to the limited number of enrolled subjects. It has been hypothesized that a cause of the age-related increase in serum adiponectin is a decrease in sex hormones with age in men; however, the interplay between testosterone and adiponectin still requires further research, including research involving both in vivo and in vitro models [37,38].

It has also been hypothesized that physical exercise exerts its beneficial effects through the regulation of adiponectin, making this adipokine an interesting molecular target. Previous studies have found an increase in this protein after several weeks of training programs [18,19,20,21]. In the present study, we observed an inverse relationship between baseline adiponectin and VO_2peak_ and Power_peak_ parameters in the entire population. Within the participants divided by age, we found a significant negative correlation between baseline adiponectin and Power_peak_ in YAs, while this correlation did not reach statistical significance in the MA group. We believe this is attributable to the small sample size. To mitigate the potential confounding effect of age, adjustments were implemented in our analysis. Even after controlling for age, significant correlations persisted between baseline adiponectin levels and both Power_peak_ and VO_2peak_. These findings indicate that the observed associations between adiponectin and exercise performance parameters are robust and not solely attributable to the effect of age. Moreover, the results suggest a decrease in baseline adiponectin levels as exercise capacity increases, indicating the adaptation of adipose tissue. Future studies are needed to delve deeper into the mechanisms underlying the relationship between exercise-induced physiological adaptations and the corresponding changes in adiponectin levels.

Additionally, we found an increase in adiponectin levels from baseline to 15 min post-exercise that directly correlates with Power_peak_. Considering Power_peak_ as a measure of exercise capacity, these findings suggest that the subjects who attained larger Power_peak_ values during the exercise protocols tend to exhibit a more pronounced rise in adiponectin levels. Our findings further suggest that training is able to modify adiponectin response depending on the exercise capacity of athletes and that an increase in adiponectin levels may be a sign of positive performance outcome.

This study is not without limitations: the small sample size may have reduced the statistical power, potentially resulting in missing data; the investigations were conducted on groups of male subjects only, omitting female samples that could have shown different adiponectin regulation in response to the same acute exercise bout; biochemical and metabolic data are missing, preventing the verification of a possible correlation between adiponectin and these parameters; the sampling time was limited to 24 h; and longer timing post-exercise could have provided additional information about the duration of adiponectin modulation and, therefore, additional information about mechanisms beyond such regulation.

On the other hand, the novel aspect of this study relates to the study population: most of the studies in the existing literature analyzed groups of people with metabolic disorders (e.g., obesity and diabetes) and assessed adiponectin at baseline and after a period of follow-up, predominantly investigating adiponectin in association with metabolic disorders. In this study, we focused on the change in adiponectin and HMW oligomer levels in well-trained subjects in relation to performance outcomes after a single bout of exhaustive exercise. Importantly, to our knowledge, this is the first study to specifically report on changes in the expression of HMW oligomers among those with greater levels of physical activity and in relation to exercise.

In summary, we have demonstrated that a single bout of exhaustive exercise evokes a significant, rapid, and lasting increase in adiponectin serum levels, particularly due to an increase in HMW oligomers. Adiponectin is associated with VO_2peak_ and Power_peak_ levels, highlighting a relationship between its regulation and exercise capacity. Further studies are needed to elucidate molecular mechanisms beyond adiponectin regulation, as well as its functional role in response to acute exercise.

## Figures and Tables

**Table 1:** Baseline characteristics of participants.

	Young Adults (N.15)	Middle-Aged Adults (N.10)	*p*-Value
Age, yrs	25.3 ± 4.1	54.0 ± 5.9	<0.001 *
Height, m	1.79 ± 0.06	1.81 ± 0.05	0.404
Weight, kg	75.6 ± 8.2	77.3 ± 7.8	0.824
BMI, kg/m^2^	23.5 ± 2.3	23.6 ± 2.4	0.782
FM, %	11.7 ± 6.0	16.9 ± 6.5	0.035 *
HR_peak_, bpm	188 ± 10	168 ± 14	0.001 *
VO_2peak_, mL/kg/min	48.4 ± 6.8	44.2 ± 5.0	0.157
Power_peak_, W	290 ± 59	329 ± 25	0.030 *

Values are reported as means ± SD. BMI, body mass index; FM, fat mass; HR_peak_, peak heart rate; VO_2peak_, peak oxygen uptake; Power_peak_, peak power. Boldface indicates a significant *p*-value (*p* < 0.05). * indicate statistically significant differences.

**Table 2:** Pearson’s correlations between baseline adiponectin levels and age, body composition, and physical exercise parameters in the entire population (25 participants).

	Correlations		Age-Adjusted Correlations	
	Baseline Adiponectin Level (µg/mL)		Baseline Adiponectin Level (µg/mL)	
	*r*	*p*-Value	*r*	*p*-Value
Age, years	0.161	0.441	-	-
BMI, kg/m^2^	−0.188	0.367	−0.193	0.365
FM, %	0.192	0.359	0.135	0.534
VO_2peak_, mL/kg/min	−0.495	0.012 *	−0.474	0.019 *
Power_peak_, W	−0.507	0.010 *	−0.645	<0.001 *

BMI, body mass index; FM, percentage of fat mass; VO_2peak_, peak oxygen uptake; Power_peak_, peak power. Boldface indicates a significant *p*-value (*p* < 0.05). * indicates statistically significant differences.

**Table 3:** Pearson’s correlations between baseline adiponectin levels and age, body composition, and physical exercise parameters in 15 young adults.

	Correlations		Age-Adjusted Correlations	
	Baseline Adiponectin Level (µg/mL)		Baseline Adiponectin Level (µg/mL)	
	*r*	*p*-Value	*r*	*p*-Value
Age, years	−0.237	0.396	-	-
BMI, kg/m^2^	−0.188	0.502	−0.200	0.493
FM, %	0.106	0.706	−0.188	0.519
VO_2peak_, mL/kg/min	−0.487	0.066	−0.498	0.070 *
Power_peak_, W	−0.662	0.007 *	−0.651	0.012 *

BMI, body mass index; FM, percentage of fat mass; VO_2peak_, peak oxygen uptake; Power_peak_, peak power. Boldface indicates a significant *p*-value (*p* < 0.05). * indicates statistically significant differences.

**Table 4:** Pearson’s correlations between baseline adiponectin levels and age, body composition, and physical exercise parameters in 10 middle-aged adults.

	Correlations		Age-Adjusted Correlations	
	Baseline Adiponectin Level (µg/mL)		Baseline Adiponectin Level (µg/mL)	
	*r*	*p*-Value	*r*	*p*-Value
Age, years	0.211	0.558	-	-
BMI, kg/m^2^	−0.243	0.499	−0.257	0.505
FM, %	0.205	0.571	0.142	0.716
VO_2peak_, mL/kg/min	−0.395	0.259	−0.347	0.360
Power_peak_, W	−0.516	0.127	−0.768	0.016 *

BMI, body mass index; FM, percentage of fat mass; VO_2peak_, peak oxygen uptake; Power_peak_, peak power. Boldface indicates a significant *p*-value (*p* < 0.05). * indicates statistically significant differences.

## Data Availability

The data presented in this study are available upon reasonable request from the corresponding author. The data are not publicly available due to restrictions in data privacy.

## Supplementary Materials

The following supporting information can be downloaded at https://www.mdpi.com/article/10.3390/biomedicines12081743/s1: Figure S1.

## References

[1] Qiu Y., Fernández-García B., Lehmann H.I., Li G., Kroemer G., López-Otín C., Xiao J. (2023). Exercise sustains the hallmarks of health. J. Sport Health Sci..

[2] Rusanova O.M., Huang Z. (2022). Cardiorespiratory System in the Context of Regular Exercise in Kayaking. Phys. Act. Health.

[3] Cabral-Santos C., Gerosa-Neto J., Inoue D.S., Panissa V.L., Gobbo L.A., Zagatto A.M., Campos E.Z., Lira F.S. (2015). Similar Anti-Inflammatory Acute Responses from Moderate-Intensity Continuous and High-Intensity Intermittent Exercise. J. Sports Sci. Med..

[4] Wadley A.J., Chen Y.W., Lip G.Y., Fisher J.P., Aldred S. (2016). Low volume-high intensity interval exercise elicits antioxidant and anti-inflammatory effects in humans. J. Sports Sci..

[5] Pedersen B.K. (2023). From the discovery of myokines to exercise as medicine. Dan. Med. J..

[6] Neto J.C., Lira F.S., de Mello M.T., Santos R.V. (2011). Importance of exercise immunology in health promotion. Amino Acids.

[7] Mika A., Macaluso F., Barone R., Di Felice V., Sledzinski T. (2019). Effect of Exercise on Fatty Acid Metabolism and Adipokine Secretion in Adipose Tissue. Front. Physiol..

[8] Knudsen N.H., Stanya K.J., Hyde A.L., Chalom M.M., Alexander R.K., Liou Y.H., Starost K.A., Gangl M.R., Jacobi D., Liu S. (2020). Interleukin-13 drives metabolic conditioning of muscle to endurance exercise. Science.

[9] Forti L.N., Van Roie E., Njemini R., Coudyzer W., Beyer I., Delecluse C., Bautmans I. (2017). Effects of resistance training at different loads on inflammatory markers in young adults. Eur. J. Appl. Physiol..

[10] Cox A.J., Pyne D.B., Saunders P.U., Callister R., Gleeson M. (2007). Cytokine responses to treadmill running in healthy and illness-prone athletes. Med. Sci. Sports Exerc..

[11] Mallardo M., Daniele A., Musumeci G., Nigro E. (2024). A Narrative Review on Adipose Tissue and Overtraining: Shedding Light on the Interplay among Adipokines, Exercise and Overtraining. Int. J. Mol. Sci..

[12] Khoramipour K., Chamari K., Hekmatikar A.A., Ziyaiyan A., Taherkhani S., Elguindy N.M., Bragazzi N.L. (2021). Adiponectin: Structure, Physiological Functions, Role in Diseases, and Effects of Nutrition. Nutrients.

[13] van Andel M., Heijboer A.C., Drent M.L. (2018). Adiponectin and Its Isoforms in Pathophysiology. Adv. Clin. Chem..

[14] Obata Y., Yamada Y., Takahi Y., Baden M.Y., Saisho K., Tamba S., Yamamoto K., Umeda M., Furubayashi A., Matsuzawa Y. (2013). Relationship between serum adiponectin levels and age in healthy subjects and patients with type 2 diabetes. Clin. Endocrinol..

[15] Tomono Y., Hiraishi C., Yoshida H. (2018). Age and sex differences in serum adiponectin and its association with lipoprotein fractions. Ann. Clin. Biochem..

[16] Vilarrasa N., Vendrell J., Maravall J., Broch M., Estepa A., Megia A., Soler J., Simón I., Richart C., Gómez J.M. (2005). Distribution and determinants of adiponectin, resistin and ghrelin in a randomly selected healthy population. Clin. Endocrinol..

[17] Staiger H., Tschritter O., Machann J., Thamer C., Fritsche A., Maerker E., Schick F., Häring H.-U., Stumvoll M. (2003). Relationship of serum adiponectin and leptin concentrations with body fat distribution in humans. Obes. Res..

[18] Moghadasi M., Mohebbi H., Rahmani-Nia F., Hassan-Nia S., Noroozi H., Pirooznia N. (2012). High-intensity endurance training improves adiponectin mRNA and plasma concentrations. Eur. J. Appl. Physiol..

[19] Mallardo M., D’Alleva M., Lazzer S., Giovanelli N., Graniero F., Billat V., Fiori F., Marinoni M., Parpinel M., Daniele A. (2023). Improvement of adiponectin in relation to physical performance and body composition in young obese males subjected to twenty-four weeks of training programs. Heliyon.

[20] Racil G., Ben Ounis O., Hammouda O., Kallel A., Zouhal H., Chamari K., Amri M. (2013). Effects of high vs. moderate exercise intensity during interval training on lipids and adiponectin levels in obese young females. Eur. J. Appl. Physiol..

[21] Saunders T.J., Palombella A., McGuire K.A., Janiszewski P.M., Després J.P., Ross R. (2012). Acute exercise increases adiponectin levels in abdominally obese men. J. Nutr. Metab..

[22] Mansouri M., Keshtkar A., Hasani-Ranjbar S., Soleymani Far E., Tabatabaei-Malazy O., Omidfar K., Larijani B. (2011). The impact of one session resistance exercise on plasma adiponectin and RBP4 concentration in trained and untrained healthy young men. Endocr. J..

[23] Varady K.A., Bhutani S., Church E.C., Phillips S.A. (2010). Adipokine responses to acute resistance exercise in trained and untrained men. Med. Sci. Sports Exerc..

[24] Aktaş H.Ş., Uzun Y.E., Kutlu O., Pençe H.H., Özçelik F., Çil E.Ö., Irak L., Altun Ö., Özcan M., Özsoy N. (2022). The effects of high intensity-interval training on vaspin, adiponectin and leptin levels in women with polycystic ovary syndrome. Arch. Physiol. Biochem..

[25] Nigro E., Sangiorgio D., Scudiero O., Monaco M.L., Polito R., Villone G., Daniele A. (2016). Gene molecular analysis and Adiponectin expression in professional Water Polo players. Cytokine.

[26] Missaglia S., Tommasini E., Vago P., Pecci C., Galvani C., Silvestrini A., Mordente A., Tavian D. (2023). Salivary and serum irisin in healthy adults before and after exercise. Eur. J. Transl. Myol..

[27] Tommasini E., Missaglia S., Vago P., Galvani C., Pecci C., Rampinini E., Bosio A., Morelli A., Bonanomi A., Silvestrini A. (2024). The time course of irisin release after an acute exercise: Relevant implications for health and future experimental designs. Eur. J. Transl. Myol..

[28] Wagner D.R., Gibson A.L., Liguori G. (2021). Health-Related Physical Fitness Testing and Interpretation. ACSM’s Guidelines for Exercise Testing and Prescription.

[29] Bouassida A., Chamari K., Zaouali M., Feki Y., Zbidi A., Tabka Z. (2010). Review on leptin and adiponectin responses and adaptations to acute and chronic exercise. Br. J. Sports Med..

[30] Bobbert T., Wegewitz U., Brechtel L., Freudenberg M., Mai K., Möhlig M., Diederich S., Ristow M., Rochlitz H., Pfeiffer A.F. (2007). Adiponectin oligomers in human serum during acute and chronic exercise: Relation to lipid metabolism and insulin sensitivity. Int. J. Sports Med..

[31] Kraemer R.R., Aboudehen K., Carruth A., Durand R., Acevedo E., Hebert E. (2003). Adiponectin responses to continuous and progressively intense intermittent exercise. Med. Sci. Sports Exerc..

[32] Jamurtas A.Z., Theocharis V., Koukoulis G., Stakias N., Fatouros I.G., Kouretas D., Koutedakis Y. (2006). The effects of acute exercise on serum adiponectin and resistin levels and their relation to insulin sensitivity in overweight males. Eur. J. Appl. Physiol..

[33] Numao S., Suzuki M., Matsuo T., Nomata Y., Nakata Y., Tanaka K. (2008). Effects of acute aerobic exercise on high-molecular-weight adiponectin. Med. Sci. Sports Exerc..

[34] Ando D., Hosaka Y., Suzuki K., Yamagata Z. (2009). Effects of exercise training on circulating high molecular weight adiponectin and adiponectin oligomer composition: A randomized controlled trial. J. Atheroscler. Thromb..

[35] Nishida Y., Higaki Y., Taguchi N., Hara M., Nakamura K., Nanri H., Imaizumi T., Sakamoto T., Shimanoe C., Horita M. (2019). Intensity-Specific and Modified Effects of Physical Activity on Serum Adiponectin in a Middle-Aged Population. J. Endocr. Soc..

[36] Kelly K.R., Blaszczak A., Haus J.M., Patrick-Melin A., Fealy C.E., Solomon T.P., Kalinski M.I., Kirwan J.P. (2012). A 7-d exercise program increases high-molecular weight adiponectin in obese adults. Med. Sci. Sports Exerc..

[37] ELsaied M.A., Masallat D., Abdel-Hamid I.A. (2019). Correlation of Adiponectin with Testosterone in Patients with and Without Type 2 Diabetes and Erectile Dysfunction. Am. J. Mens Health.

[38] Mancuso P., Bouchard B. (2019). The Impact of Aging on Adipose Function and Adipokine Synthesis. Front. Endocrinol..

