# Supplementary figures and images for: Effects of Exhaustive Exercise on Adiponectin and High-Molecular-Weight Oligomer Levels in Male Amateur Athletes
Source: Biomedicines. 2024 Aug 2;12(8):1743. doi: 10.3390/biomedicines12081743 (PMC11351654; doi:10.3390/biomedicines12081743)

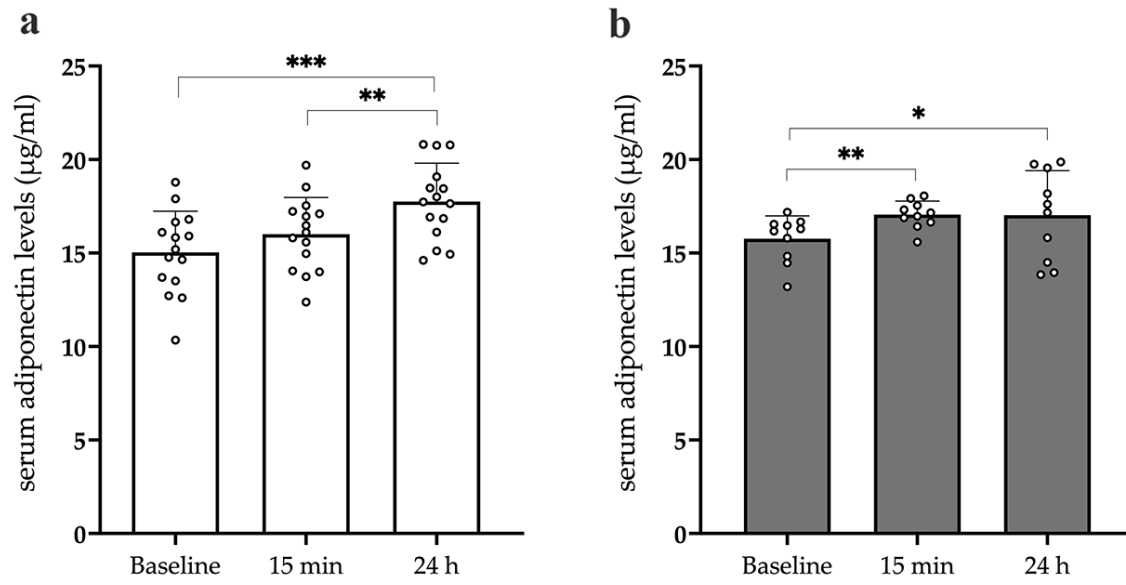

Figure S1.

Supplement: Supplementary file 1 [file biomedicines-12-01743-s001.zip › biomedicines-3089985-Supplementary.pdf]
